# Increased neural responsiveness to distractors irrespective of perceptual load explains attention deficit in post‐stroke fatigue

**DOI:** 10.1111/jnp.70002

**Published:** 2025-06-24

**Authors:** Annapoorna Kuppuswamy, Anthony Harris, William De Doncker, Adrian Alexander, Nilli Lavie

**Affiliations:** ^1^ Department of Clinical and Movement Neuroscience, Institute of Neurology University College London London UK; ^2^ School of Biomedical Sciences, Faculty of Biological Sciences University of Leeds Leeds UK; ^3^ Institute of Cognitive Neuroscience University College London London UK; ^4^ Queensland Brain Institute The University of Queensland St Lucia Australia; ^5^ San Fernando General Hospital, South West Regional Health Authority San Fernando Trinidad and Tobago

**Keywords:** attention, distractor, perceptual load, post‐stroke fatigue, visual perception

## Abstract

Post‐stroke fatigue (PSF) is a prevalent symptom associated with attention deficits. However, it is currently unclear what drives these. Here we applied Load Theory of Attention to investigate the role of perceptual load in the relationship between attention, distraction and fatigue levels in PSF. Thirty‐two chronic stroke survivors performed a selective attention task of either low, medium or high perceptual load (varied through the number of relevant target features and their combinations). Neural responses to targets and distractor checkerboard flicker (vs. no flicker) were measured with frequency‐tagged EEG responses. The results showed that fatigue severity scores were predictive of response slowing, and that this slowing was increased with higher levels of perceptual load. Fatigue severity was also associated with increased neural responsiveness to distractors, specifically: EEG 10 Hz (distractor flickering frequency) power was greater in the presence (vs. absence) of distractor flicker for participants with high (vs. low) fatigue‐symptoms scores, across all levels of perceptual load in the later time period of each task trial. Overall, these results clarify the exacerbating effects of perceptual load on fatigue‐related slowing, stressing the importance of cognitive, as opposed to purely motoric, deficits. Importantly, they demonstrate that increased fatigue severity involves reduced ability to suppress neural responses to irrelevant distractors, irrespective of perceptual load on attention. An account for attention in PSF based on a specific deficit in distractor suppression that is found irrespective of task demands can explain a myriad of PSF symptoms (e.g. sensory perceptual overload, difficulties to concentrate).

## INTRODUCTION

Post‐stroke fatigue (PSF) is a prevalent and persistent symptom after stroke characterized by severe debilitating exhaustion, both physically and mentally (Barbour & Mead, 2012; Whitehead et al., 2016). People with high levels of PSF report increased fatigue when performing cognitively demanding activities (Johansson & Rönnbäck, 2012) as well as problems concentrating (Radman et al., 2012), and often complain that they are unable to filter out irrelevant background stimuli. For example, they cannot carry a conversation in a crowded environment (e.g. a party), and find visual clutter overwhelming, resulting in avoidance of public spaces (Barbour & Mead, 2012; Eilertsen et al., 2013; Young et al., 2013). These are suggestive of an attention deficit, however, previous research of attention in PSF has led to mixed results. While some studies clearly indicate deficits in the ability to sustain attention over time (Radman et al., 2012; Ulrichsen et al., 2020), as well as in selective attention and ‘executive control’ over irrelevant distraction (Carrick et al., 2024; Johansson & Rönnbäck, 2012; Radman et al., 2012), others reported generalized cognitive impairment expressed in slower processing speed and some memory deficits, but no impairment in selective attention and executive control (Graber et al., 2019; Maaijwee et al., 2014; Pihlaja et al., 2014; Schepers et al., 2006). The lack of consistency in attentional tests across studies, and absence of measures of associated neural activations, make it difficult to draw conclusions about the nature of attention deficit that can explain the lived experience of PSF. Here we applied the Load Theory framework of selective attention and cognitive control (Lavie, 2005; Lavie et al., 2004, 2014) to clarify the nature of attention deficit in PSF, while testing the derived predictions for neural activity with EEG.

Load theory framework provides an attention model that clarifies the roles of cognitive executive control and perceptual capacity limits in determining attention. In this model, cognitive executive control serves to direct attention focus to stimuli according to their task relevance and maintain attention focus on task‐relevant, rather than task‐irrelevant, stimuli throughout task performance. Cognitive control over processing in accordance with task‐relevance is necessary for a selective attention focus, and much research has demonstrated increased irrelevant distraction during task performance when cognitive control is loaded with another task (e.g. when a visual attention task is carried under high working memory load (Brand‐D'Abrescia & Lavie, 2008; de Fockert et al., 2001; Lavie et al., 2004; Lavie & De Fockert, 2005) or during multi‐tasking (Brand‐D'Abrescia & Lavie, 2008; Lavie et al., 2004)).

However, while necessary, cognitive control is in itself insufficient to prevent distractions, as long as the tasks involve low perceptual load. Much research has demonstrated that task situations of low perceptual load (e.g. with just one target stimulus: a letter or a colour patch, in the Flanker (Eriksen & Eriksen, 1974) or Stroop (Stroop, 1935) tasks, respectively) result in distractor processing, despite a fully functioning executive control that is available to control attention during the task processing (i.e. in single task conditions of low working memory load (Brand‐D'Abrescia & Lavie, 2008; de Fockert et al., 2001; Lavie et al., 2004; Lavie & De Fockert, 2005)). This is because in load theory perceptual processing is automatic and proceeds on all stimuli (including distractors) that are within the person's capacity, until it runs out of neural resources (Bruckmaier et al., 2020; Lavie, 2005; Lavie et al., 2004). Therefore, while executive cognitive control is necessary to direct attention to relevant stimuli, it cannot prevent a ‘spillover’ of neural resources to irrelevant distractors, when these are within the person's capacity.

The level of demand on perceptual capacity (i.e. the perceptual load of a task) is thus a critical determinant of the ability to focus attention selectively on relevant information, and successfully ignore irrelevant distractors, in addition to a fully operative executive cognitive control function. Support for this claim has come from an abundance of demonstrations that irrelevant distractors cannot be ignored and elicit neural responses and behavioural interference effects, in tasks of low perceptual load (e.g. single‐feature search tasks, for example, search for any red shape) but their processing is diminished with tasks of high perceptual load that exhausts perceptual capacity (e.g. ‘conjunction search’ whereby the target is defined by feature combinations, for example, a red square or a green circle, among non‐targets that also include the opposite shape‐colour combinations among other target‐similar items; Bahrami et al., 2007; Bruckmaier et al., 2020; Carmel et al., 2011; Lavie, 2005; Rees et al., 1997; Schwartz et al., 2005; Torralbo et al., 2016). Thus, increased perceptual load results not only in increased neural activity related to the attended task stimuli but also in reduced neural response to unattended, task‐irrelevant distractors. Importantly, this load‐induced ‘neural resource trade‐off’ (Torralbo et al., 2016) has a positive outcome of reduced distractor interference effects on behaviour.

Functional spectroscopy measures of cerebral oxygen metabolism levels further demonstrate that as metabolism levels related to attended stimuli rise with increased perceptual load in the task, metabolism levels related to unattended stimuli drop. A tight temporal correlation between the load effects of both increased metabolism in attended processing and decreased metabolism in unattended processing points to the well‐known brain‐wide limits on oxygen metabolism as the source of limited perceptual capacity (Bruckmaier et al., 2020). The latter finding is of particular relevance to research establishing that PSF involves a systemic reduction in oxygen metabolism levels (Klinedinst et al., 2019) since neural computations heavily depend on oxygen metabolism, this would suggest that PSF involves reduced perceptual processing capacity.

Thus, drawing on this PSF research, together with findings of increased fatigue developing in the course of performance in attention‐demanding tasks (Johansson & Rönnbäck, 2012), as well as the complaints of PSF sufferers about over‐stimulation, we hypothesized that attention deficits in PSF may involve reduced perceptual capacity. This hypothesis leads to the prediction that smaller increases in the level of perceptual load in a task will be sufficient to exhaust the more limited perceptual capacity in those who suffer from PSF compared to those who do not. Importantly, since exhausting perceptual capacity in task‐relevant processing under high load conditions has the benefit of reducing irrelevant distraction, then the hypothesis of reduced perceptual capacity in PSF also leads to predicting that lower levels of perceptual load in the task will be sufficient to reduce the processing of irrelevant distractors in PSF compared to control.

Alternatively, if attentional deficits in PSF are mainly due to reduced executive cognitive control capacity (see (Carrick et al., 2024; Johansson & Rönnbäck, 2012; Radman et al., 2012)), this would be expected to lower their ability to actively maintain task‐relevance control of attention, thus resulting in an increase in distractor processing overall (i.e. irrespective of the level of perceptual load in the task). Of course, PSF may involve a reduction in both perceptual capacity and executive cognitive control abilities. If this was the case, we predict that the failure to prioritise the task in the face of distractors in PSF would result in particularly harmful effects of distraction in tasks of higher perceptual load. This is because people with PSF would experience an overload with the increased distractor processing (due to their reduced executive control over distractor interference) combined with reduced perceptual capacity being more taxed by increased perceptual load.

To test these hypotheses, we used a selective attention task involving a perceptual load manipulation and measuring neural responses to the task stimuli and to irrelevant distractors and tested stroke patients, experiencing different levels of PSF. To manipulate perceptual load, we used a well‐established perceptual load task involving feature versus conjunction search in a temporal stream of crosses of various colours and orientations (Bahrami et al., 2007; Bruckmaier et al., 2020; Jacoby et al., 2012; Klinedinst et al., 2019; Schwartz et al., 2005). Under the low load condition, targets were defined based on a single feature (stimulus colour), whereas in the high load condition, a conjunction of both colour and orientation features needed to be identified. We have also included a novel condition presumed to involve an intermediate load level, in which targets were defined based on two separate features (two different colours). We reasoned that the added requirement to perceive two features (instead of one) would constitute only a moderate level of increase in perceptual load, so that this condition may allow a sensitive measure of a more subtle reduction in perceptual capacity in PSF. The task was performed either in the presence or absence of visual distractors. Neural responses to the task stimuli and distractors were measured with electroencephalography (EEG) using a ‘frequency tagging’ method. Frequency tagging is a well‐used and robust method for tracking the allocation of attention (Morgan et al., 1996). This method capitalizes on the fact that the steady‐state visual evoked neural response to a flickering stimulus increases in amplitude with increased attention to the stimulus (Toffanin et al., 2009). This method has been previously used to measure neural responses to distractors in the same perceptual load paradigm as that used here (Jacoby et al., 2012). In addition, since previous research has indicated that PSF involves deficits in sustained attention (Ulrichsen et al., 2020) we have also considered the effects of time on task (potentially leading to task‐fatigue) on neural processing of both the attended stream and the distractors.

We thus set out to investigate interactions between attention, distraction, perceptual load and fatigue in chronic stroke using a selective attention task with frequency tagged EEG responses to targets and distractors, and considering the effects of time on task.

## METHODS

### Participants

Following written informed consent in accordance with the Declaration of Helsinki, 32 stroke survivors (Table 1) participated in this cross‐sectional observational study approved by the London Bromley Research Ethics Committee (REC reference number: 16/LO/0714). Inclusion criteria: (1) first‐time ischaemic or haemorrhagic stroke, (2) stroke >3 months at time of testing (to ensure participants are medically stable and past the sub‐acute phase when stroke survivors adjust to their new post‐stroke state). Exclusion criteria were (1) any other neurological disorder, (2) use of anti‐depressants or centrally acting medication, (3) clinically depressed or Hospital Anxiety and Depression Scale (HADS) scores ≥11, (4) sensory impairment or neglect and (5) hand grip strength and nine‐hole peg test (NHPT) of the affected hand <60% of unaffected hand (Table 2).

**TABLE 1:** Clinical presentation of the study participants.

	Mean (SD)	Spearman *ρ*	*p*‐value
PSF severity (FSS‐7)	3.22 (1.99)		
State fatigue (VAS)	3.25 (2.29)	.68	<.0001
Age (years)	60.97 (12.49)	−.15	.4092
Grip (% unaffected hand)	94.27 (17.96)	−.00	.9801
NHPT (% unaffected hand)	89.72 (23.05)	−.28	.1229
Depression—HADS	4.69 (3.52)	.08	.6680
Anxiety—HADS	4.56 (3.16)	.25	.1633

*Note*: Data indicate the mean and SD of the various fatigue, motor, cognitive and mood scores. Spearman correlations were used to assess the association between FSS‐7 and all continuous measures. The spearman ρ and associated *p*‐values indicate the relationships between FSS‐7 score and the respective measures. There is a significant positive association between trait (FSS‐7) and state fatigue. Non‐significant relationship between FSS‐7 and all measures indicate that the group studied suffered from what is known as ‘primary’ fatigue, that is, that which is not secondary to obvious motor or mood impairments. Mean grip and NHPT scores of approximately 90% reflects the minimal impairment of the group.

Abbreviations: HADS, Hospital Anxiety and Depression Scale; NHPT, nine hole peg test.

**TABLE 2:** Stroke characteristics across all study participants.

Stroke survivors total	32
Hemisphere affected	
Right	13 (40.62%)
Left	19 (59.38%)
Stroke type	
Ischaemic	29 (90.62%)
Haemorrhagic	3 (9.38%)
Vascular territory affected	
ACA	0 (0.00%)
MCA	23 (71.88%)
PCA	2 (6.25%)
Brainstem/Cerebellum	7 (21.88%)
Time post‐stroke (years)	
Mean (SD)	4.60 (3.52)

*Note*: Information taken from the discharge summary hospital notes of each individual stroke survivor. Lesion location and hemisphere affected were defined based on the vascular territory affected by the stroke. The mean and SD of the time post‐stroke, taken in years, from the time of the stroke to the time of participation in the study, indicate that the studied cohort were all chronic stroke survivors.

Abbreviations: ACA, anterior cerebral artery; MCA, middle cerebral artery; PCA, posterior cerebral artery.

### Measures

PSF severity was assessed by requesting participants to fill in the Fatigue Severity Scale‐7 (FSS‐7) (Johansson et al., 2014) which is the standard instrument for measuring fatigue in stroke as well as in a wide range of chronic disorders with fatigue as a symptom (Johansson et al., 2014; Kleinman et al., 2000; Learmonth et al., 2013; Lerdal et al., 2011). This measure asks about fatigue experienced in the week leading up to the day of testing and has high internal validity and reliability (Lerdal & Kottorp, 2011). Fatigue on the day of testing was also measured using visual analogue scale (VAS) where participants responded to a single statement ‘How tired are you right now on a scale of 0–10?’

### Stimuli and apparatus

The experiment was run on a PC with a 24‐inch monitor, at a screen resolution of 1280 × 768 and a refresh rate of 60 Hz. Participants were seated 70 cm from the monitor and made their responses using a standard USB keyboard. The experiment was controlled using the Psychophysics Toolbox for Matlab (Brainard, 1997; Kleiner et al., 2007).

**FIGURE 1:**
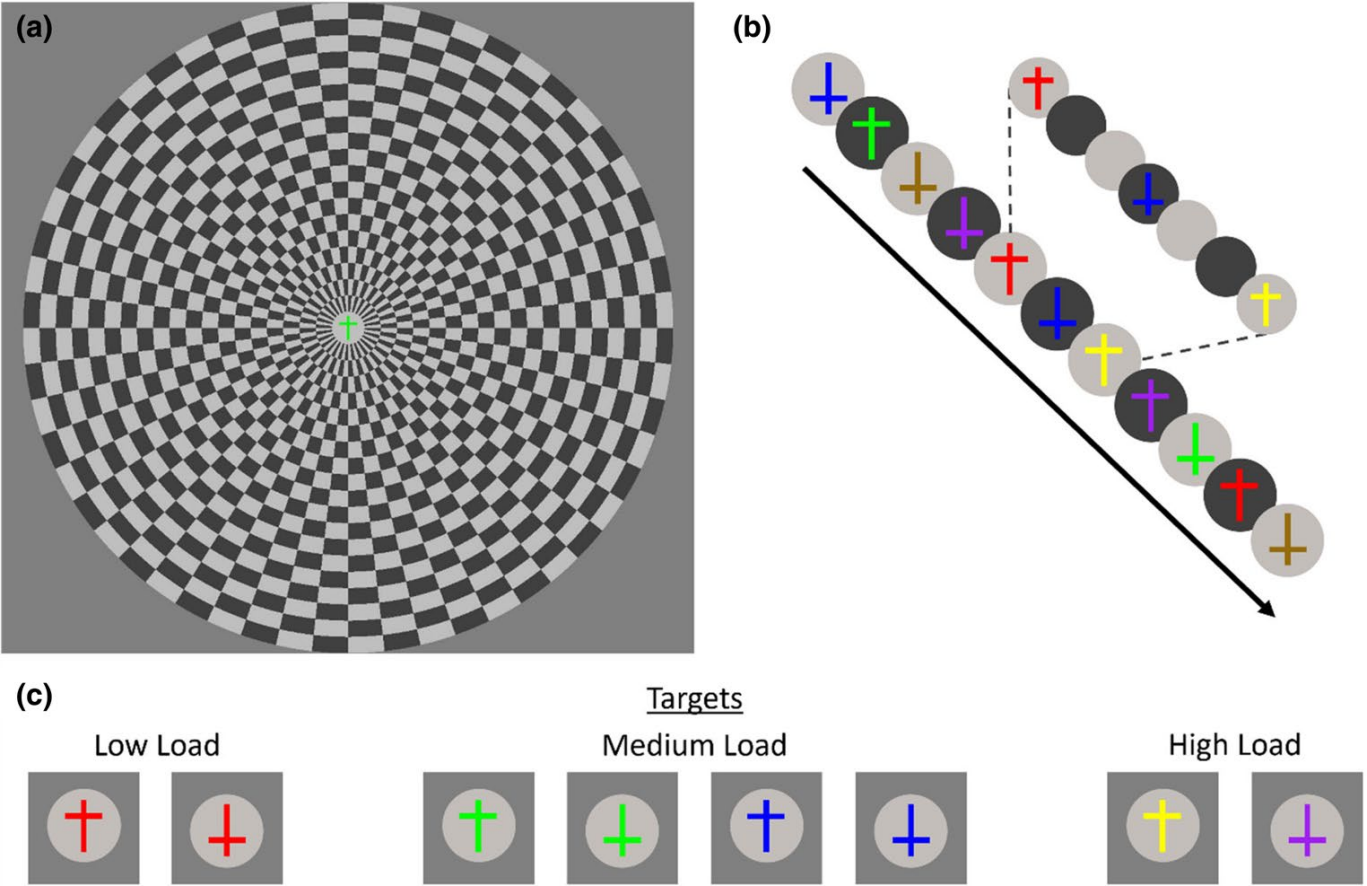
Attention paradigm. (a) Participants attended a central stream of crosses, responding when condition‐specific targets were detected. In the Flicker condition, they were required to ignore a peripheral 10 Hz flickering checkerboard presented around the central target stream. This checkerboard was absent in the No flicker condition. (b) The central stream contained upright and inverted crosses that varied among six different colours. These crosses were presented for 250 ms each, followed by a 500 ms blank interval in which the target pedestal continued to flicker at 4 Hz. Each stream lasted 24 s, containing a total of 32 crosses (4 targets, 28 non‐targets). (c) The targets for each perceptual load condition. Participants responded to any red cross in the low load condition, any green or blue cross in the medium load condition and specifically to upright yellow crosses and inverted purple crosses in the high load condition.

The experimental design was a full crossing of three perceptual load conditions (low, medium and high load) with two peripheral stimulation conditions (no flicker vs. flicker), with the flickering peripheral stimulation functioning as distractors in this task. In all conditions, participants were presented with streams of 32 coloured crosses (2.5° tall, 1.5° wide, .3° thick), with each stream constituting a trial from one of the six conditions. Each cross could appear in one of six colours and each was either upright or inverted (Figure 1b). The colours were as follows: red (RGB: 255, 0, 0), green (RGB: 0, 255, 0), blue (RGB: 0, 0, 255), brown (RGB: 156, 102, 31), yellow (RGB: 255, 255, 0) and purple (RGB: 160, 32, 240). For upright and inverted crosses, the horizontal bar was .4° above or below the midline, respectively. All stimuli were presented on a grey background (RGB: 127, 127, 127). Each cross was presented for 250 ms, followed by a 500 ms inter‐stimulus interval (ISI) To achieve a strong readout of focused attention on the task streams, on all trials the attended task stimuli were presented on a central disk (2.5° × 2.5°), flickering between dark grey (RGB: 64, 64, 64) and light grey (RGB: 192, 192, 192) at a frequency of 4 Hz (one change every 250 ms), with a cross presented on every third disk. The presentation of this flickering disk at a different frequency to the frequency of cross appearance (1.33 Hz) allowed us to measure attention to the task relevant stream, independent of any influence from the properties of the specific task stimuli. On Flicker trials, the central disks were surrounded by a ring of unattended counterphasing visual checkerboards (Figure 1a). These checkerboards spanned from 2.5° to 14° into the periphery, divided into 19 concentric rings of .6° each and were made up of 56 radial sections, each spanning 6.4° of radial angle. Successive cells of this checkerboard were each alternately coloured light or dark grey and their colours counterphased in a square‐wave fashion at a rate of 10 Hz. On No‐flicker trials, this checkerboard was replaced by the grey background colour. Such checkerboards elicit strong neural responses across a range of visual areas (Schwartz et al., 2005) at the precise frequency of the visual flicker (Jacoby et al., 2012).

### Procedure

Three levels of perceptual load were included (Figure 1c). In the low load condition, the targets were upright and inverted red crosses. In the medium load condition, targets were any (upright or inverted) green or blue cross. In the high load condition, targets were upright yellow crosses and inverted purple crosses.

At the beginning of each trial, participants were shown a display that indicated their targets for that trial with both a text description and an image of each target. This display remained on the screen for 3 s and then a fixation point appeared for 1 s, followed by the trial stimuli. Participants fixated on a central plus sign (.3° × .3°), and when the stream of stimuli appeared, and a target was detected, participants responded by pressing the spacebar. Participants were required to withhold responses to all non‐target stimuli. Each trial contained four targets which included any of the crosses pre‐specified for each load (e.g. low load targets would be red crosses two inverted and two upright, whereas for medium load trials, targets were both upright and inverted versions of the green and purple crosses), and 28 non‐targets selected from the set of all remaining stimuli. The trial duration was 24 s. The position of targets in the trial was restricted such that the first two items could not be targets, nor could the last item, and no two targets could successively appear directly following each other.

Participants completed three practice trials that were identical to the experiment trials (one trial selected for each load level, in random order), then 48 trials (of 32 crosses each) of the main task. These 48 experiment trials were composed of eight trials for each of the six different conditions (three load conditions crossed with two flicker conditions). The order of all different trial types was random. Participants received a self‐paced break after every 10 trials of the main task.

### EEG recording

Whole‐scalp EEG data were recorded using a 64‐channel cap array (ActiCap, Herrsching, Germany) in accordance with the 10–20 international EEG electrode array and a BrainAmp EEG amplifier system (BrainProducts, Gilching, Germany). During online recordings, channels FCz and AFz were used as the reference and ground, respectively. Vertical and horizontal electrooculogram (EOG) recordings were used to capture horizontal eye movements and blinks. The EEG signal was sampled at 1 kHz and event markers were sent from the stimulus presentation PC to the BrainAmp amplifier via the TriggerBox with millisecond precision.

### EEG preprocessing

Using a combination of EEGLAB (Delorme & Makeig, 2004) and custom Matlab scripts, data were imported, and bad channels were identified using EEGLAB's default kurtosis‐based automated procedure. The average number of identified channels was 2.5 (SD = 1.88), and no participant had any of the analysed channels rejected (see below). Any bad channels were then interpolated using spherical spline interpolation followed by re‐referencing against the grand average of all scalp electrodes. Epochs were extracted from 2 s after the beginning of each trial until 1 s before the end of each trial at 23 s and baseline corrected against the average voltage for the entire epoch. Steady‐state visual evoked potential (SSVEP, a.k.a., ‘frequency tag’) amplitudes were calculated across the trial period by means of a Fast Fourier Transform with .5 Hz resolution on each 1‐s period of trial data to produce 21 temporal bins within each trial. The SSVEP responses to the background flicker (10 Hz frequency) as well as the frequency of flicker at the target location (4 Hz) were baseline corrected by subtracting the mean of the 10 neighbouring frequencies, excluding the two nearest frequencies and the two most extreme values (see Rossion et al., 2020, for justification of the method). Relevant harmonics were identified by computing the *z* score of the harmonic frequency (20 Hz for the background flicker and 8 Hz for the target flicker. No higher harmonics were identified) relative to the surrounding noise frequencies. Harmonics that exceeded a one‐tailed *z*‐threshold of 1.64 (i.e. *p* < .05) were summed with the principal frequency.

### Statistical analyses

Spearman rank correlations were used to identify associations between PSF severity as measured with FSS‐7 and state fatigue as measured with VAS, age, grip strength, NHPT, HADS–Depression and HADS–Anxiety and Time Post‐Stroke. Wilcoxon rank sum tests were used to identify the association between FSS‐7 score and categorical measures of sex, hemisphere affected, type of stroke and vascular territory affected.

**FIGURE 2:**
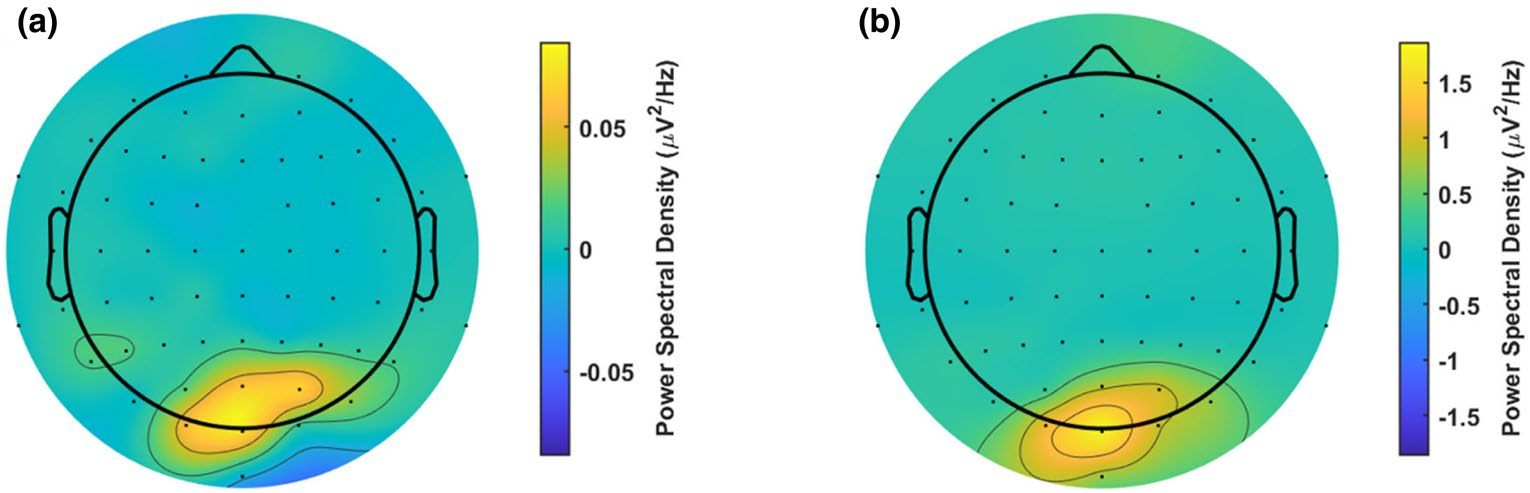
Topographies of 4 Hz and 10 Hz responses. (a) Power spectral density at the 4 Hz target frequency across the scalp and (b) PSD for the 10 Hz responses. Both panels show a posterior locus of the effect.

Based on past research that used a very similar design and analysis (Jacoby et al., 2012), neural analyses were focused on the average response from electrodes Oz, O1 and O2. Visualization of the SSVEP response across the scalp showed this location corresponded with the location of strongest SSVEPs in our study (Figure 2). Linear mixed effects analyses were used to investigate the relationship between the effects of perceptual load, flicker, FSS‐7 scores, within‐trial‐time, trial number and our measures of reaction time, 4 and 10 Hz power. One outlier participant was excluded from the EEG analyses as their oscillatory power values were an order of magnitude stronger than the other participants. Accuracy was assessed with a generalized linear mixed effects model with probit link, using the same predictors. Each linear mixed effects model included a random effect of participant. VAS was not included in these models due to its high correlation with FSS‐7 (see Results), which may cause problems with multicollinearity. FSS‐7 scores were included in the model in their continuous form but were subject to a median split for the purposes of visualization (Figure 4).

We employed a model selection procedure in which each combination of factors was assessed, and the supported model was the one with the lowest Bayesian Information Criterion (BIC). BIC is a metric that expresses degree of support for a model after penalizing for model complexity. BIC was used, rather than the Akaike Information Criterion (AIC) as AIC tends to underpenalize model complexity and to select complicated, difficult to interpret and models. In practice, the two criteria selected the same model in almost every case. Lower BIC values indicate more support for the model. BIC differences of 2–5 indicate a moderate difference, and differences of 5 or greater indicate a substantial difference. When including a factor in the model, its main effect and all possible interactions with other included factors were included. As this procedure makes, it difficult for a main effect to survive the model selection if a given factor does not interact with any other factor, for each winning model we also compared its BIC to all iterations of the same model with each possible main effect included that was not included in the initial model.

## RESULTS

### Stroke characteristics

Table 1 shows the clinical presentation of study participants. The median FSS‐7 score in females was 5.14 (IQR = 2.95), whereas the median FSS‐7 score in males was 1.9 (IQR = 3.5), but this trend did not reach significance (Wilcoxon *Z* = 1.55, *p* = .06, *r* = .34). Spearman rank correlations between PSF severity (FSS‐7) and all continuous clinical measures are shown in Table 1. These revealed a significant positive association between PSF severity and fatigue on the day of testing (VAS) (Spearman *ρ* = .68, *p* < .001). No other variables correlated with PSF severity.

Table 2 shows stroke characteristics of the study participants. There was no difference in FSS‐7 score between right [median = 4.5 (IQR = 4.2)] and left hemisphere [median = 1.9 (IQR = 3.0)] strokes (*p* = .4, effect size *r* = .14), ischaemic [median = 1.9 (IQR = 3.7)] and haemorrhagic [median = 2.8 (IQR = 2.0)] strokes (*p* > .999, *r* = .01), and MCA [median = 2.8 (IQR = 4)], PCA [median = 3.8 (IQR = 1.9)] and brainstem/cerebellar [median = 1.9 (IQR = 2.6)] strokes (*p* = .6, effect size *η* ^2^ = −.04). There was also no correlation between PSF severity and time post‐stroke (Spearman *r*_s_ = .25, *p* = .17).

### Reaction time

**FIGURE 3:**
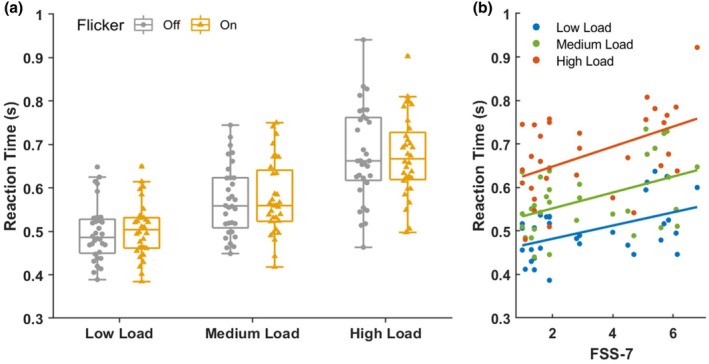
Reaction time results. (a) The time taken to respond to a target stimulus for each load condition, in the presence (yellow) or absence (grey) of a flickering surround. RT was significantly longer as load increased, with no difference across flicker conditions. Small dots and triangles represent individual participants. (b) Reaction times were significantly slower in those reporting high trait fatigue, and this pattern was exacerbated at high perceptual load.

Of the family of linear mixed effects models used to predict the single‐trial reaction time data, the supported model incorporated only the fixed effects of fatigue and perceptual load, indicating there was no effect of the flicker (vs. no flicker) on participants' response times (Figure 3a). The effect of fatigue was marginal (*β* = .01, *p* = .058), but the effect of perceptual load (*β* = .08, *p* < .001) and the fatigue × load interaction (*β* = .003, *p* = .003) were both significant (Figure 3b). The BIC values showed a relatively small preference of this model over the next best model, which incorporated only the effect of load (ΔBIC = .72), however, the AIC values showed substantial support for the inclusion of fatigue in the model (ΔAIC = 13.98). These results indicate that as both fatigue and load increased, responses were slowed, and that the slowing effect of fatigue was exacerbated at high load.

### Accuracy

The generalized linear mixed effects analysis predicting detection of targets on single trials supported the null model that incorporated only the random effect of participants and no other predictors (ΔBIC vs. next best model = 9.57).

### 4 Hz power (target frequency)

Linear mixed effects analysis of brain responses at the target frequency supported a model (ΔBIC vs. next best model = 9.81) in which 4 Hz power was predicted only by the fixed effect of flicker (*β* = −.12, *p* < .001). Addition of the each of the other fixed effects led to an increase of the BIC of the model (load: ΔBIC = 10.07; fatigue: ΔBIC = 8.82; trial number: ΔBIC = 5.31; within‐trial time: ΔBIC = 8.83). Thus the 4 Hz target responses were weaker when the surrounding flicker was present, but were equivalent across all other factors.

### 10 Hz power (distractor frequency)

**FIGURE 4:**
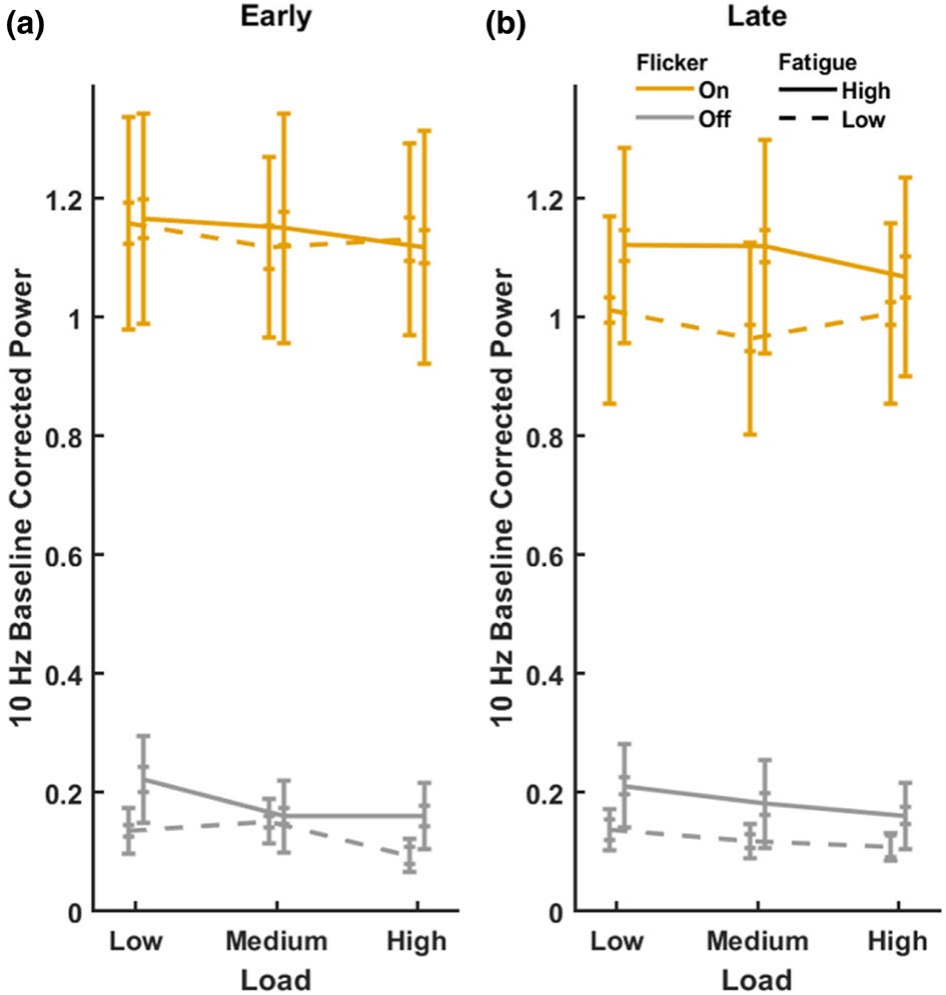
Ten Hertz power by condition. Ten Hertz power at each level of perceptual load and flicker, separately for low‐ and high‐fatigue participants (based on a median split). The data are shown separately for the early period (the first 11 s) within the trial (a) and the late period (the last 10 s) in the trial (b). Note that error bars reflect both between‐subjects standard error of the mean (SEM; outer fins) for comparing between low‐ and high‐fatigue participants, and within‐subjects SEM (inner fins) for all other comparisons.

Linear mixed effects analysis of brain responses at the distractor frequency supported a model (ΔBIC vs. next best model = .72) that included the factors fatigue, flicker, and within‐trial time (Figure 4). The model revealed significant effects of flicker (*β* = 1.02, *p* < .001) and within‐trial time (*β* = .01, *p* = .004), and significant flicker × within‐trial time (*β* = −.01, *p* < .001) and fatigue × flicker × within‐trial time (*β* = .002, *p* = .008) interactions (Figure 4). To clarify the pattern of the three‐way interaction, we divided the data into an early period comprised of the first 11 within‐trial times and a late period comprised of the final 10 within‐trial times, and analysed the fixed effects of fatigue and flicker within each of these time periods. As shown in Figure 4, the early time‐points contained an effect of flicker (*β* = .96, *p* < .001) but no effect of fatigue and no fatigue × flicker interaction (*p*s > .123). At the later time‐points, however, the significant effect of flicker (*β* = .79, *p* < .001) was supplemented with a significant fatigue × flicker interaction (*β* = .03, *p* < .001), with no fixed effect of fatigue (*p* = .618). No other effects or interactions were significant (all *p* > .145). Addition of the fixed effect of perceptual load further improved the fit of the model (ΔBIC = −13.72). The effect of perceptual load was significant (*β* = −.02, *p* < .001). None of the other effects had their significance or direction of effect altered by this addition. Addition of the fixed effect of trial number increased the BIC of the model (ΔBIC = 1.77).

Overall these results indicate that 10 Hz responses were stronger in the presence of 10 Hz flicker, as expected. Ten Hertz responses were also weakened by increasing perceptual load, as expected however this effect did not interact with the flicker condition, or with fatigue.

The two‐way interaction of flicker and within‐trial time suggested that the distractor effect was generally stronger in the earlier (compared to later) trial period. However, the three‐way interaction and the follow‐up analyses of the early versus late within‐trial period indicated that the attenuation of distractor response in the later trial period depended on fatigue scores, and Figure 4 clarifies that reduction in the distractor 10 Hz response in the later time period is only seen in participants with low‐fatigue scores. Thus, higher fatigue levels are associated with reduced ability to attenuate distractor processing in the course of a 24‐s trial period.

The relatively small ΔBIC for the comparison to the next‐best model indicates that the model including only the factors of flicker and within‐trial time was only marginally worse than the preferred model. This model included all the same significant effects as the preferred model, with the exception of the three‐way interaction, and was also improved by the addition of a fixed effect of load (ΔBIC = 6). However, given the strong penalization for model complexity in the BIC calculation, it is clear that although the three‐way interaction made a small contribution to the model fit, it was nevertheless sufficient to outweigh the BIC penalty over model complexity, so that it was ranked as the top fitted model. The two models here were strongly preferred relative to the next best (third‐ranked) model (ΔBIC > = 24.42).

## DISCUSSION

In 32 minimally impaired, non‐depressed, chronic stroke survivors with no visual neglect or extinction, our results show that PSF severity level is predictive of slower responses in a visual attention task. The findings also show that responses are generally slower in tasks of higher perceptual load, as expected, and that the slowing of responses with increased perceptual load was larger for participants with higher PSF levels. There was no effect of distractor interference on response times or accuracy. However, our EEG analysis revealed that 10 Hz power (the frequency of the flickering distractor) was significantly higher in the distractor flicker condition compared to the no‐flicker condition, indicating a clear neural response to the distractor. Increased levels of perceptual load diminished 10 Hz power across all participants. While this is consistent with the expected effect of perceptual load on distractor processing, the lack of interaction with distractor conditions suggests this reflected a general effect of high perceptual load resulting in suppression of endogenous alpha band power (8−12 Hz), consistent with previous reports (Gutteling et al., 2022; Molloy et al., 2015). Importantly, the 10 Hz response to the distractor flicker (vs. no flicker) was larger in participants with higher PSF levels (compared to those with lower PSF levels) in the later period of the trial stream, and this was found irrespective of the level of perceptual load in the task. Our EEG measure of attended processing was based on the 4 Hz power, the frequency at which the attended stimulus stream was presented. Although we did not observe any effect of load or fatigue on 4 Hz power, the 4 Hz power was weaker in the presence of a distractor flicker (vs. no flicker) condition, demonstrating a distractor interference effect on the neural response to attended stimuli.

Overall, the findings reflected a specific pattern of deficits in attention in PSF together with preserved abilities, as we discuss in more detail next. Our finding that PSF is associated with slower response speed is consistent with much previous research (Radman et al., 2012; Ulrichsen et al., 2020), importantly our study clarified that increased perceptual load exacerbates the fatigue‐related slowing, so that participants with higher PSF levels showed greater slowing of their response in task conditions of higher perceptual load. This finding is in support the hypothesis that fatigue involves reduced perceptual capacity. Our EEG findings also revealed increased neural processing of the distractor (10 Hz power in flicker vs. no flicker) in participants of high PSF levels, which is found in the later period of a trial stream, irrespective of the levels of perceptual load in the task. These findings suggest that the attention deficits in fatigue can be explained as reflecting an executive cognitive control failure to suppress irrelevant distractors, combined with a deficit to sustain attention throughout an experimental trial period. Interestingly the 4 Hz frequency‐tagged responses to the attended stream was of similar magnitude across PSF levels, and the distractor interference effects on the 4 Hz responses also did not differ between low‐ and high‐fatigue levels. Thus, participants with high levels of fatigue maintained an equivalent level of 4 Hz responses and accuracy levels to those of low‐fatigue levels, albeit with significantly slower performance, especially when perceptual load in the task increased. The overall pattern of these results is suggestive of a specific selective attention deficit in maintaining the suppression of distractor processing, when the demands on sustained attention are increased, rather than reflecting a simple failure in the executive cognitive control ability to direct attention appropriately to the task stimuli in the first place.

In relation to the clinical picture of PSF, the findings that increased distractor processing is found in high PSF with a prolonged task‐trial period, across all task levels of perceptual load, despite the exacerbated slowing of their responses with increased load, and while maintaining a similar magnitude of neural responses to the attended stimulus stream can explain why high PSF sufferers find it more effortful to concentrate (Johansson & Rönnbäck, 2012). It can also explain patients' reports of being overwhelmed by a visual scene that consists of several stimuli, some of which are likely to be perceived as distractors (Barbour & Mead, 2012; Young et al., 2013).

Our findings that high PSF levels are associated with an increased neural response to distractors in the absence of increased behavioural‐interference effects also points to the importance of assessing neural responses to distracting stimuli as a clearer measure of any alterations in attention due to fatigue. Furthermore, the findings that fatigue is associated with a failure to attenuate neural response to a task‐irrelevant distractor, may provide further support to recent proposals that a fundamental deficit that explains both cognitive and motor aspects of PSF, is an alteration in sensory perception (including proprioception) (Kuppuswamy, 2017, 2021, 2024). This proposal suggests that incoming sensory information (whether self‐generated in proprioception or from external task‐irrelevant stimuli) is poorly attenuated in those with high fatigue. For example, poor attenuation of proprioceptive input from contracting muscles can lead to a muscle contraction being experienced as effortful. Similarly, reduced ability to attenuate processing of task‐irrelevant visual stimuli can make a visual task feel more effortful. This should be especially the case considering that the slowing effect of perceptual load on task responses was exacerbated in participants with high PSF levels. Despite being effortful and leading to slowing of responses in cognitive tasks, previous studies of motor tasks show that there is no significant effect of PSF on performance metrics such as reaction times (De Doncker et al., 2021) and motor‐task completion (Doncker et al., 2020). These results suggest that the slowing of responses is due to a deficit in cognitive processing (perceptual in the present study). They also demonstrate a dissociation between measures of performance and reported effort. Specifically, although the patients with higher levels of chronic fatigue are able to achieve the same levels of performance and suffer the same effect of distractor interference on their neural response to the target, the greater distractor processing does entail more effort for them. In the current study, lack of modulation of distractor effects by fatigue on behavioural measures, but increased neural response to the distractor with increased severity of fatigue is in further support of the dissociation between patients’ reports about perceived effort and performance.

It is interesting to note that the effects of increased demand on sustained attention as a function of time in the task were found within each trial stream, but not as the experiment went on (c.f. previous sustained attention studies in PSF (Radman et al., 2012; Ulrichsen et al., 2020)). However, it is possible that our task trials were more demanding than previous tasks, since they involved the rapid presentation of a stream of stimuli, together with high‐contrast checkerboard distractors that were flickering on 50% of the time. It is therefore plausible that our task was more sensitive to reveal a within‐trial sustained attention deficit in distractor suppression for people with high PSF levels, rather than one that can only be found in the later experiment period.

## CONCLUSIONS

We show that attention impairment in PSF is attributed to a reduced attenuation of the neural processing of irrelevant distractors as attention is drained in the course of rapid processing stream. This attentional decline is found across all levels of perceptual load in the attended task, in the absence of increased distractor interference effects, neither on the neural processing of the attended stream nor on task performance RT and accuracy. In addition, an exacerbation of response slowing in people with high levels of PSF in tasks of high perceptual load, points to a perceptual deficit component in their response slowing. Taken together these effects support an account of attention alterations in PSF leading to both increased effects of perceptual load, and rapid draining of their executive cognitive control ability to suppress the neural response to irrelevant stimuli. Overall, our findings clarify the nature of attention impairment in PSF and can also explain the experience of cognitive fatigue, and difficulties to concentrate together with the feeling that a stimulating environment might be overwhelming for the large group of PSF sufferers.

## AUTHOR CONTRIBUTIONS

**Annapoorna Kuppuswamy:** Conceptualization; resources; writing – review and editing; writing – original draft; funding acquisition. **Anthony Harris:** Conceptualization; writing – review and editing; writing – original draft; data curation; methodology; visualization. **William De Doncker:** Data curation; project administration; investigation. **Adrian Alexander:** Investigation. **Nilli Lavie:** Conceptualization; resources; writing – review and editing; writing – original draft.

## FUNDING INFORMATION

This study has been funded by the Wellcome Trust 202346/Z/16/Z. AMH is supported by the Australian Research Council (DE220101019).

## CONFLICT OF INTEREST STATEMENT

There are no conflicts of interest to report by any of the authors.

## Data Availability

The data that support the findings of this study are available from the corresponding author upon reasonable request.
